# What Are the Occupational Risks in Forestry? Results of a Long-Term Study in Slovakia

**DOI:** 10.3390/ijerph16244931

**Published:** 2019-12-05

**Authors:** Martin Jankovský, Michal Allman, Zuzana Allmanová

**Affiliations:** 1 Department of Forestry Technologies and Construction, Faculty of Forestry and Wood Sciences, Czech University of Life Sciences, Kamýcká 129, 165 00 Praha 6—Suchdol, Czech Republic; 2 Department of Forest Harvesting, Logistics and Ameliorations, Faculty of Forestry, Technical University in Zvolen, T.G. Masaryka 24, 960 53 Zvolen, Slovakia; Michal.allman@tuzvo.sk (M.A.); Zuzana.allmanova@tuzvo.sk (Z.A.)

**Keywords:** safety management systems, forest harvesting, occupational accidents, incidence rate

## Abstract

Temporal patterns in occupational safety and health can shed light on the efficiency of safety measures companies adopt and identify when workers are prone to occupational accidents. We analyzed these patterns to identify the effects of factors such as the share of salvage logging, experience, age, daytime, weekday, and more on the number of occupational accidents at Forests of the Slovak Republic (FSR). We analyzed a database of 2963 occupational accidents and 443 occupational illnesses suffered by FSR employees and contractors. We then analyzed a subset of said database, containing 401 accident records coded according to European Statistics at Work manual. We used regression and correlation analyses and generalized linear models to test the relationship between the accident frequency and volume of harvested timber and volume of salvage logging. We used logistic regression, chi2 tests, and Cramér’s V statistic to test when accidents occur within shifts, weeks, and months. We found the volume of harvested timber significantly affects the frequency of severe and fatal accidents of contractors (R 0.81; *p* < 0.05), whereas, for employees, the relationship was insignificant. Over time, the number of accidents and incidence rate decreased, and inexperienced or older workers were the most prone to accidents.

## 1. Introduction

Forestry is regarded as a dangerous industry [1]. Ozden et al. (2011) [2] state that all countries that provide incidence rate statistics by industry report the highest rates in forestry. Regardless of the use of modern techniques and machinery, occupational accidents are frequent and, in many cases, fatal, especially in forest harvesting [3,4]. These accidents are often followed by material and environmental damage, as well as financial compensations to the victims or their families [5], thus having additional effects on the financial stability of businesses in forestry.

The most demanding occupations in forestry connect to motor–manual forest harvesting (chainsaw felling and tree processing, skidding with a skidder). It is a hard work and requires holding awkward positions [6]. In addition, workers are exposed to a combination of factors stemming from the work environment or the processed materials [7]. The main risk factors connected to the work environment include rugged terrain, climatic conditions, biological agents, and exposure to noise, vibrations, and exhaust fumes. Other risks connected to forest harvesting include using sharp, powered tools, heavy loads, and heavy machinery [8,9,10]. Due to this, workers also often suffer from various occupational diseases of the muscular, skeletal, nervous, vascular, and auditory systems [11,12,13].

As [14] state, felling and extraction of timber are operations where most fatal accidents occur. Each of these operations has its specifics, depending on the natural and technological conditions and share of manual labor in the timber production process. Timber logging, namely motor–manual, is connected with a high risk of an occupational accident (OA) occurring [15]. On the other hand, mechanized logging is considered relatively safe, though with the deployment of cut-to-length technologies, new risks emerge, connected with their increased complexity and mental strain of the operators [16,17,18].

From a temporal point of view, accidents in forest harvesting occur mostly in the first half of the workweek. According to [15,19,20], most OAs occur on Mondays and Tuesdays, though [15] reported a rather uniform distribution, with the exception of Friday, when the frequency of OAs is approximately half of the frequency in other workweek days. Within the work shifts, the likelihood of a worker to suffer an accident peaks before the lunch break, followed by a smaller increase before the end of the shift [15,19,21,22]. There are several potential causes for such a distribution of accidents throughout the shifts: (i) worker effort increases in the times when most accidents occur; (ii) the dose of work environment factors (e.g., noise, vibration, heat/cold exposure) exceeds the capacity of the workers to cope with them; (iii) fatigue sets in; (iv) workers deplete their energy and fluid reserves from meals, thus increasing the fatigue level, etc. [15,23,24,25,26,27].

Slovak forestry faces the problem of a large share of salvage logging, ranging from 31% in the year 2000 to 57% in the year 2015 [28]. This was caused by windstorms in the early 2000s and a subsequent bark beetle outbreak in the country [28,29]. These problems are the most visible when we look at the largest raw timber producer in the country—Forests of the Slovak Republic, GOE (government-owned enterprise; FSR). The company manages about 46% of all Slovak forests (886,252 ha) and harvests about half of all timber (4.7 million m^3^ in the year 2015) [28]. The large share of salvage logging can further increase the number of occupational accidents, including, but not limited to, forest harvesting [15,30,31].

This paper focuses on assessing the occupational accidents suffered by the employees and contractors of the FSR during the 2000–2017 period, from a temporal and operational point of view. We analyze the records of the FSR and test the effects of several factors on the number of accidents or the severity. We set the following hypotheses for testing: (H1) contractors of the FSR suffer significantly more severe and fatal OAs than employees; (H2) the total annual volume of harvests and the share of salvage logging significantly affect the number of OAs occurring at the FSR within a given year; (H3) inexperienced workers suffer significantly more OAs than experienced workers; (H4) worker age significantly affects the number of OAs; (H5) significantly more OAs occur during the first half of the workweek; (H6) significantly more OAs occur during the first half of the work shift; (H7) significantly more OAs occur in forest harvesting than in any other occupation; (H8) the distribution of types of injuries vary significantly throughout the year (e.g., more fractures occur in a given season); and (H9) the distribution of body parts injured vary significantly throughout the year (e.g., more lower-limb injuries in a given season).

## 2. Materials and Methods

### 2.1. Data Sources

During our analyses, we used databases of the FSR OSH (Occupational Safety and Health) department. According to the OSH management system of the FSR, all OAs suffered by the company employees and contractors were stored in a generic human resources database. From the year 2006 on, FSR kept a comprehensive database of all severe and fatal OAs according to the Slovak regulation no. 500/2006 Coll. [32]. Until that year, record-keeping was managed by individual branches. The regulation mandates that every OA must be registered by the company and for OAs that cause the injured employee to lose working days, a record must be kept in accordance with the template specified by the regulation. The template specified in [32] transposed the regulation of the European Parliament and Council no. 89/391/EEC [33] to Slovak legislation. Therefore, the Slovak OA record template contained (among other information) a narrative mode of injury, description of the material agent, and cause of accident. These characteristics were then coded by FSR safety officers according to the European Statistics on Accidents at Work (ESAW) [34]. In cases when the narrative parts of the record conflicted with the information encoded through [34], we resolved the conflict by recoding the ESAW variables according to the information provided in the narratives. This database only contained information on the OAs that occurred to workers with a direct relationship with the FSR —employees (E), contractors (C), and their employees, and people who harvest timber allotments from the FSR forests (F) (FSR sells them the right to harvest a small amount of low-grade timber for firewood). It did not contain any information on the OAs that occurred to subcontractors.

### 2.2. Variables Analyzed

From the generic database on human resources for the years 2000–2017, we gathered information on the number of employees and the occupational structure, as well as general information on the number and structure of OAs, based on the relationship of the worker with the FSR. In case of FSR employees, we recorded: (i) number of employees (v_1_) divided by sex—male or female and according to type of work—technical/office staff or manual worker; (ii) number of OAs (v_2_) classified by severity —minor (no days lost), registered (up to three days lost), severe (more than three days lost), and fatal; (iii) number of days lost due to an OA or an occupational illness (OI) (v_3_); (iv) reported near-misses (v_4_); (v) number of OIs (v_5_)—vasoneurosis, borreliosis, hearing loss, and other. Workers who were not employees of the FSR, i.e., contractors and people who harvest their timber allotments were obliged to report to the FSR only severe and fatal OAs. In these cases, we gathered the same information as for employees.

The more-detailed database created from OA records was effectively a subset of the generic human-resource database. The OA record database contained data on 401 cases which occurred during the years 2006–2017. We gathered the following information from the OA records: sex (v_6_), age (v_7_), occupation (v_8_), and experience (v_9_) of the injured worker, as well as the date (v_10_), hour (v_11_), day of week (v_12_), month (v_13_), severity (v_14_), operation (v_15_) during which the incident occurred, type of injury (v_16_), and injured body part (v_17_).

### 2.3. Data Analyses

We included all OA records in the database of the FSR OSH department. To achieve a more robust dataset, we merged similar occupations (e.g., all employees of the directorate and branches who worked inside offices were labeled “office”, workers in the garage were labelled “mechanic”, etc.). We analyzed the following occupations: (i) animal handler; (ii) office; (iii) feller; (iv) forester; (v) mechanic; (vi) haulage truck driver; (vii) wood processing line operator; (viii) wood processing line supervisor; (ix) forest worker (various manual tasks); (x) other (unspecified); (xi) skidder operator; (xii) machine operator (unspecified); (xiii) silviculture worker (various manual tasks in nurseries, forest establishment, tending, etc.); (xiv) harvester and forwarder operator; (xv) helicopter pilot; and (xvi) loader operator.

The workers in all mentioned occupations carried out operations during which they suffered an injury. Due to the variability of the operations, we merged similar operations, as well: (i) felling (including delimbing and bucking); (ii) repairs and maintenance; (iii) timber haulage; (iv) forest management; (v) skidding; (vi) wood processing (automated at a conversion depot); (vii) walking; (viii) other (unspecified); (ix) operating other machines; (x) sideline operations; (xi) silviculture; (xii) game; (xiii) animal care; and (xiv) forest road construction and maintenance. As for severity of an accident, we distinguished between minor injuries that did not cause the loss of working days, registered injuries that caused a loss of between one and three working days, severe injuries that caused a loss of more than three working days, and fatal accidents that caused death. This way, we were able to work with a more manageable dataset.

First, we calculated the Incidence Rate Coefficient (IRC) according to [1]:

(1) $$IRC=\frac{OA_{TOT}\times 200,000}{H_{TOT}}$$

where OA_TOT_ is the total number of occupational accidents and illnesses in the company, 200,000 is the constant for calculating the incidence rate per 100 employees who work standard eight-hour shifts, and H_TOT_ is the total number of hours worked by all employees of the company.

We analyzed the relationships between v_2_ (number of OAs) categorized according to v_14_ (severity of the OA) and the relationship of the worker with the FSR, the share of incidental fellings, and annual harvested volume. We used generalized linear models (GLMs) to analyze the potential difference between v_2_ (number of OAs) suffered by employees and contractors of the FSR categorized according to v_14_ (severity of the OA). We used logistic regression of the GLMs to analyze the effects of v_7_ (age), v_8_ (occupation), v_9_ (experience), and v_15_ (operation) on v_2_ (number of OAs) categorized according to v_14_ (severity of the OA) and their relationship with the FSR (employees and contractors).

We used contingency tables for basic analyses and an χ^2^ test to analyze the distribution of the variable v_2_ (number of OAs) in individual categories. We also used this approach when analyzing v_7_ (age), v_9_ (experience), v_11_ (hour), v_12_ (day of week), v_13_ (month), v_16_ (type of injury), and v_17_ (injured body part), as well as for analysis of the distribution of the fatal accidents for v_2_ (number of OAs) and v_6_ (sex). We used an χ ^2^ test, along with Cramér’s V coefficient, to test v_2_ (number of OAs) and v_7_ (age), v_11_ (hour), v_12_ (day), and v_13_ (month). Microsoft Word, Excel, Statistica 12, and R software served to process and analyze the gathered material.

## 3. Results

### 3.1. General Characteristics of the Studied Datasets

The total number of employees of the FSR decreased within the observed period. A gradual shift toward the system of contracting logging, as well as silviculture operations, which began in the year 2006, caused the decrease from 13,176 employees in the year 2000 to 3,484 in the year 2017. The biggest layoffs took place in the first six years of the observed period, when the number of employees of the company dropped by 8400 people to 4696 employees. The change of the business model resulted in a change of the employee structure, as well—the share of office staff increased from 29% in the year 2000 to 64% in the year 2017. The FSR reached the breaking point in this parameter in the year 2004, when the number of office staff exceeded the number of manual workers. Despite the shift toward outsourcing manual labor, the share of male employees was stable throughout the observed period and fluctuated around 84%.

**Figure 1:**
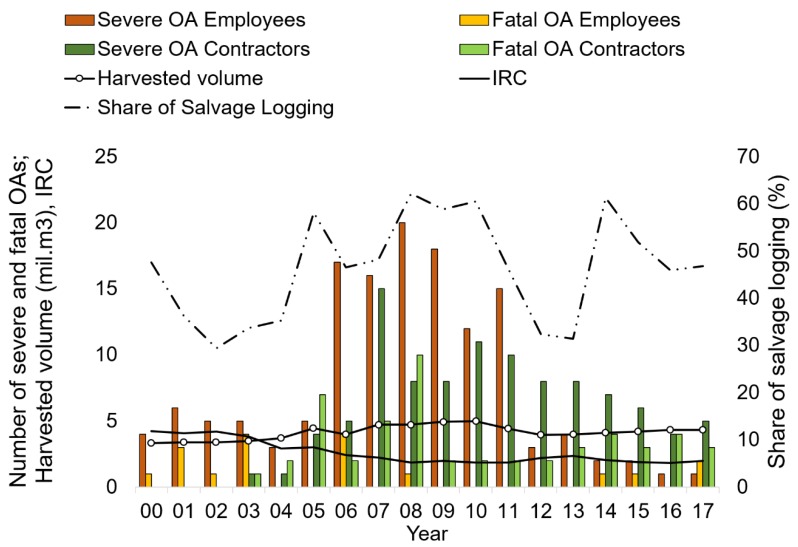
The number of severe and fatal occupational accidents (OAs) suffered by employees and contractors of the Forests of the Slovak Republic, government-owned enterprise (GOE), incidence rate coefficient (IRC), total volume of harvested timber, and the share of salvage logging in the observed period of 2000 to 2017.

In total, employees, contractors, and people who harvested timber allotments from FSR forests suffered 2,963 OAs within the observed period (Table 1). Employees suffered 2,787 OAs during the observed period, out of which 9.5% were minor injuries, 84.9% were registered, 5% were severe, and 0.6% were fatal. On average, employees suffered 164 OAs annually, ranging from 56 (2010) to 505 (2000). The employee structure was not the only cause of the OA fluctuation, as the IRC also fluctuated, though it gradually decreased (Figure 1). Contractors suffered 153 OAs registered by the FSR. Out of these, 66% were severe and 34% were fatal. Those who harvested their wood allotments reported 23 OAs, out of which 26% were severe and 74% were fatal (difficult to conceal). Contractors reported 27% fewer severe accidents and 288% more fatal accidents than FSR employees. The differences between the number of OAs suffered by FSR employees from those of contractors were statistically significant for all severity categories (Table 2).

Besides the OAs, FSR employees suffered 443 OIs in the observed period, i.e., 26 annually. The leading OI was borreliosis (47.6%), followed by vasoneurosis (44.7%), noise-related illnesses (4.8%), and other (2.9%). The FSR started recording the number of days lost due to OAs or OIs systematically after the year 2006, when Slovakia updated its OA record template. Starting from that year, until 2017, FSR employees lost 39,375 days due to OAs or OIs, i.e., 3,281 annually or 46.7 per OA or OI. We also found that the workers were reluctant to report near-miss incidents. In total, they reported 22 instances during the observed period, ranging from zero to six instances per year.

The total volume of timber harvested by the FSR rose from 3.3 to 5.0 million m^3^ (Figure 1), including wood allotments and standing timber sales. This was due to the increasing volume of salvage logging (mean share of salvage logging throughout the observed period was 49%). The relationship between the number of severe OAs and the share of salvage logging was not statistically significant. On the other hand, the total volume of harvested timber had a moderately strong relationship with the number of severe OAs suffered by employees (*R* = 0.55; *p* < 0.05). The number of fatal accidents did not have a statistically significant relationship with both the share of salvage logging and total volume of harvested timber. For contractors, the number of severe OAs correlated strongly with the annual volume of harvest (*R* = 0.81; *p* < 0.05), though the share of salvage logging did not show a statistically significant relationship. However, both factors showed a significant moderate relationship with the number of fatal accidents for contractors (Table 3).

### 3.2. Temporal Aspects of Occupational Accidents

We observed the highest incidence rate among employees in the year 2000 (4.23 within group), when the company started its transformation and the smallest in the year 2016 (1.84 within group). The incidence rate per 100 employees showed that manual workers were almost nine times more likely to suffer and OA than office staff.

**Figure 2:**
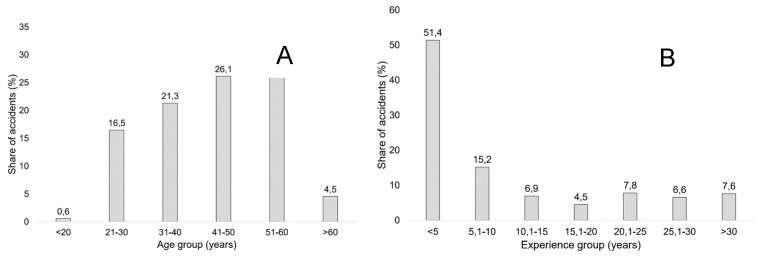
Distribution of occupational accidents according to experience (**A**) and age (**B**) of workers.

The ages at which employees and contractors suffered an OA throughout the observed period were diverse (Figure 2a). Though older workers aged between 51 and 60 years were the most prone to suffer an OA (31% of all OAs), a much younger class of 21- to 30-year-olds followed as the second most prone to suffer an accident. An χ^2^ test of OA distribution showed that the differences between the numbers of OAs suffered by age classes were statistically significant (χ^2^ 152.44 > 11.1, df = 5, *p* = 0.00), though age did not affect the severity of the sustained injuries (χ^2^ 9.69 < 18.3, df = 10, *p* = 0.47; Cramér’s V = 0.12).

The OA distribution according to experience (Figure 2b) showed that inexperienced workers (less than five years of experience) suffered the most OAs, followed by workers with five to ten years of experience. The differences between the number of OAs suffered by particular experience classes were statistically significant, as proven by the χ^2^ test (χ^2^ 339.8 > 12.6, df = 6, *p* = 0.00). However, Cramér’s V of 0.24 showed that the relationship between the experience of workers and the distribution of accidents they suffered was weak. Similarly, the χ^2^ test and Cramér’s V showed a weak relationship between the experience of the workers and the severity of the accidents they suffered (χ^2^ = 33.88 > 21.0, df = 12, *p* = 0.00; Cramér’s V = 0.24).

**Figure 3:**
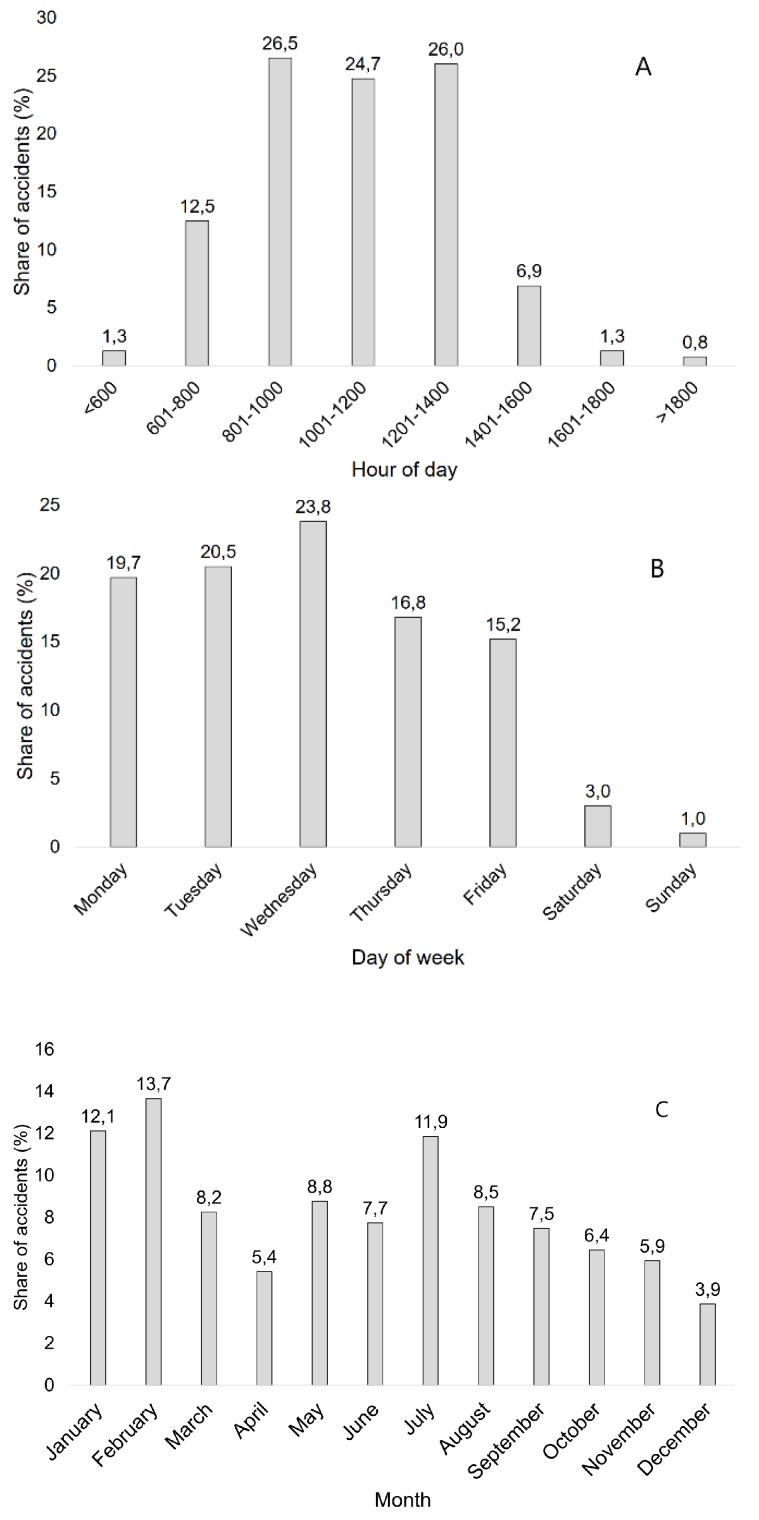
Distribution of occupational accidents: day of week (**A**), hour of day (**B**), and month (**C**).

Most of the workers worked a standard Monday-to-Friday workweek, though, in some specific occupations (harvester operators, logging truck drivers, processing line operators, game management, and animal handling), they worked during the weekends too. In the OA record data set, the number of OAs occurring during the weekends was small. The number of OAs (Figure 3a) gradually increased from Monday to Wednesday and then decreased until the end of the week. Moreover, accidents were more frequent in the first two days of the workweek than on Thursday and Friday. Despite this, the day-wise differences in OA frequency were not statistically significant (χ^2^ = 9.23 < 9.5, df = 4, *p* = 0.06) throughout the Monday-to-Friday workweek. The severity of the OA did not relate to the day of the workweek either (χ^2^ = 15.43 < 21.0, df = 12, *p* = 0.22; Cramér’s V = 0.14).

Like the workweek schedule, shift work was not standard for workers and only applied to specific occupations (same as those listed in the previous paragraph). Those workers worked 12-h shifts, starting from 6:00 a.m. until 6:00 p.m. Most other workers, however, worked a standard eight-hour shift. The OA frequency varied throughout the shifts (Figure 3b). Two peaks are visible: the first in the 8:01–10:00 a.m. slot and the second in the 12:01–2:00 p.m. time slot. Sixty-five percent of accidents occurred before noon. The hourly OA distribution showed significant differences (χ^2^ = 298.16 > 14.1, df = 7, *p* = 0.00), and we also found a significant, though weak, relationship between the distribution of OAs during the day and the severity of the accident (χ^2^ = 37.63 > 23.7, df = 14, *p* = 0.00; Cramér’s V = 0.22).

In the OA record dataset, the most dangerous season, considering the frequency of the accidents, was winter, when 29.2% of all OAs occurred (Figure 3c). Namely, in January and February, OAs were frequent. Summer proved to be a dangerous season, as well. Twenty-eight per cent of all OAs occurred during summer, culminating in July. The fewest OAs occurred in December. The differences between the monthly OA frequency were significant (χ^2^ = 40.82 > 19.67; df = 11; *p* = 0.00). However, the χ^2^ test and Cramér’s V showed no significant relationship between the month and OA severity (χ^2^ = 12.37 < 33.92; df = 22; *p* = 0.95; Cramér’s V = 0.13).

The season proved to have little to no effect on the type of injury sustained during the work, as the χ^2^ test proved there were no significant differences in the distribution of OAs throughout the year (χ^2^ = 20.30, df = 27, *p* = 0.82). Table 4 shows that, overall, the most frequent type of injury was fractures, followed by superficial injuries and lacerations, and strains, sprains, and dislocations. While fractures and superficial injuries and lacerations were the most abundant in winter, sprains, strains, and dislocations occurred mostly during summer.

Similarly, the season had no effect on the injured body parts (χ^2^ = 21.91, df = 21, *p* = 0.40). Out of all cases registered in the detailed database, most abundant were injuries of lower extremities, followed by injuries of upper extremities (Table 5). Season-wise, most injuries of the extremities (both upper and lower) were sustained by the victims in the summer, while most injuries of the head and neck occurred during winter. The least-injured body parts were back and spine.

### 3.3. Occupational and Operational Aspects of Occupational Accidents

Timber harvesting was the most dangerous activity; most OAs occurred while carrying out operations connected to it. Moreover, 67.6% and 16.2% of all fatal OAs occurred during felling and timber extraction, respectively. The most endangered group of workers (Table 6) were fellers, who suffered 28% of all OAs, as well as 71.1% of all fatal OAs. The logistic regression showed that there was indeed a significant relationship between being a feller and the probability of sustaining a fatal accident (*p* < 0.05). The fellers were followed by machine maintenance and repairs. Timber haulage was also a dangerous occupation. However, we have to keep in mind that the FSR outsources 94.3% of all logging and only 48.4% of timber haulage.

From the logistic regression analysis, it is apparent that fatal OAs suffered by FSR employees were affected by multiple operations, as well as the experience of the employees. Age proved to be an insignificant factor for fatal OAs. Severe OAs were affected by factors such as experience, age, and occupation. Similarly, the occurrence of registered OAs was also affected by employee age, their experience, and occupation.

### 3.4. Fatal accidents

**Figure 4:**
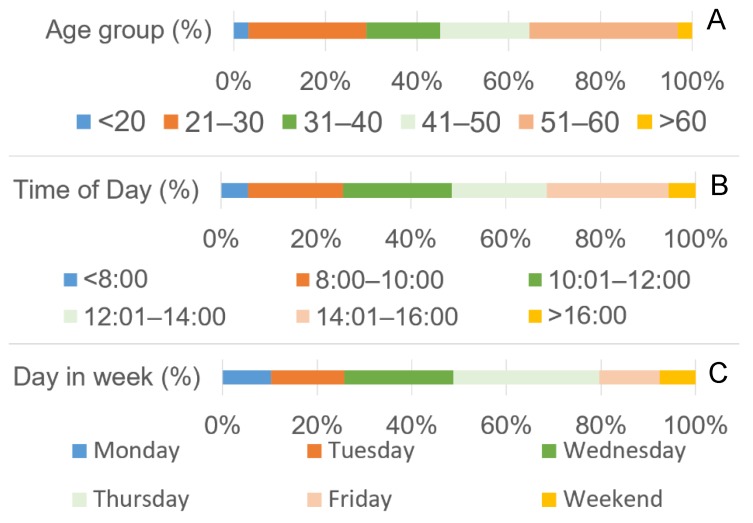
Distribution of fatal accidents according to age (**A**), throughout the work shifts (**B**), and throughout the week (**C**).

**Figure 5:**
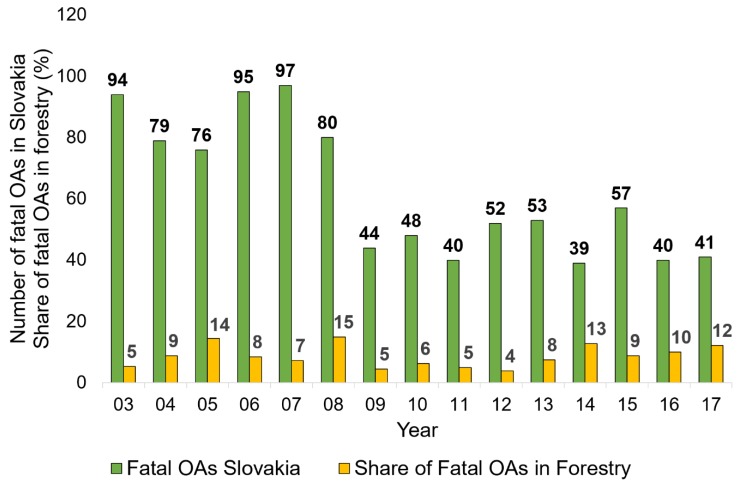
Number of all fatal accidents occurring in Slovakia and the share of fatal accidents in forestry in the period of 2003 to 2017.

Workers aged between 51 and 60 years suffered the most fatal OAs, followed by workers in the 21–30 group (Figure 4a). A χ^2^ test proved that the fatal OAs distribution was uneven between the age groups (χ^2^ = 11.08 > 11.07, df = 5, *p* = 0.05). Regarding the time of day, the distribution was more or less uniform in the two-hour segments from 8:00 a.m. to 4:00 p.m. The most fatal OAs occurred between 2:01 and 4:00 p.m., followed by the time slot between 10:01 a.m. and 12:00 p.m. (Figure 4b). Regarding the day in week, almost one-third of all fatal OAs occurred on Thursday, followed by Wednesday. Interestingly, almost 8% of all fatalities occurred during the weekend, despite how work was only occasionally carried out on Saturday or Sunday (Figure 4c). The share of fatal occupational accidents in forestry and overall in Slovakia ranged from 4% to 15% registered in 2008, with an average percentage of 8.7% (Figure 5). Though the OA frequency was inconsistent, the variability throughout the shifts was not statistically significant (χ^2^ = 5.98 < 11.07, df = 5, *p* = 0.31).

## 4. Discussion

The problem of occupational safety and health was studied by numerous authors. Tsioras et al. (2011) report that the accident rate declined from 155 accidents per million hours worked (years 1980–1989) to 77 accidents (years 2000–2009) in the Austrian federal forest enterprise. Bentley et al. (2005) state that the accident rate decreased in New Zealand, as well, from 20 accidents per million hours worked in 1999 to 14 accidents in 2001. The decreasing accident rate could relate to improvements to the safety management systems in forestry enterprises and promoting safety programs in the industry [35,36,37]. We observed a similar trend considering the employees of FSR, when both the absolute numbers of OAs and IRC decreased, from 505 OAs in the year 2000 to 62 in the year 2017 and 3.88 IRC in the year 2000 to 1.80 IRC in the year 2017.

Two thirds of the OAs reported occurred to workers between the ages of 25 and 54 years, with the mean age reaching 43 years. Wilhelmson et al. (2005) [38] report similar results: that older workers in forestry, between the age of 50 and 59, suffer the most OAs. In addition, Tsioras et al. (2014) [15] report the mean age of the workers who suffered an OA was 40 years. Lagerstrom et al. (2017) [39] and Laschi et al. (2016) [8] report that the workers in the age group of 31–50 years suffer the most OAs; furthermore, they report that the duration of the sick leave increases with age. This could be due to the fact that older workers tend to lose vigilance during the shifts, especially when the shifts are prolonged and nonstandard [40]. Another group suffering many accidents was composed of young, inexperienced workers. Inexperienced workers frequently lack the skills and habits necessary to work safely, so they tend to get injured more frequently [41]. The high share of accidents occurring to young workers points to the industry’s underperformance in safety training, because, as Boini et al. (2017) [41] state, the frequency of OAs halves if the students undertake occupational health and safety training.

The number of OAs at the FSR increased during the first half of the week, from Monday until Wednesday, when almost two-thirds of all accidents occurred. From Wednesday until the end of the week, the accident frequency gradually decreased. This goes against many similar studies in forestry. For example, Lagerstrom et al. (2017) [39], Laschi et al. (2016) [8], Tsioras et al. (2014) [15], and Wigglesworth (2006) [42] all state that accidents occur on Monday most frequently, reporting that more than one-fifth of all accidents occur on this day. The differences between accident frequency during particular workweek days proved to be insignificant in our case, as approximately one-fifth of all accidents occurred on Monday and Tuesday. However, as Wigglesworth (2006) [42] states, the increasing number of accidents could be due to fatigue of workers, which accumulates throughout the week and is likely to increase in nonstandard shifts.

Many seasonal factors affect the number of accidents occurring in forestry throughout the year. Forest harvesting is most intensive during the drier summer months. This connected to the increased accident frequency during the summer season. Lagerstrom et al. (2017) [39] reported similar results, with a uniform distribution of accidents throughout the summer season. Similarly, Ghaffariyan (2016) [43] reported the largest share of accidents in Australia occurred in January and February (more than 10% each month) and lowest in December and September. On the other hand, even though harvesting is not as intensive during the winter season, the working environment is more dangerous. Tsioras et al. (2014) [15] reported the highest accident frequency in March and February. We observed that most accidents occurred during the winter months of January and February. The lowest frequency of OAs occurred in December (4%), which could be attributed to the upcoming winter holidays and the connected lowered productivity [43].

Almost two-thirds of all accidents at the FSR occurred before noon, and the frequency peaked between 8:01 and 10:00 a.m. The second period in which most accidents occurred was just after noon, between 12:01 and 2:00 p.m. According to Folkard and Tucker (2003) [44] and Folkard and Lombardi (2006) [45], the distribution of accidents throughout the day is connected to the duration and type of shift, as well as the frequency of breaks. This could explain the gradual increase of accidents occurring around noon. The decrease in accident frequency after 2:00 p.m. could be linked to gradual decrease in productivity throughout the day [46] that reaches a minimum with the approaching end of the shift (only exceptionally the shifts lasted longer than after 3:30 p.m.).

Naturally, workers exposed to diverse and demanding work environment and material agents during harvesting operations suffered the most injuries [1,26,47,48]. Felling and stem processing were the most dangerous operations; more than one-fifth of all accidents occurred during these operations. These were followed by repairs and maintenance and timber haulage. On the other hand, only about every tenth accident occurred during timber extraction. The relatively high share of injuries sustained during timber haulage compared to extraction could be due to the fact that FSR outsources a considerably smaller portion of haulage than other harvesting operations, which can affect the number of reported accidents [49]. Similar overall results were reported by Ghaffariyan (2016) [43] and Potočnik et al. (2009) [49], who also observed that majority of injuries were sustained during forest harvesting operations.

Contrary to what we expected, season had little effect on both the type of injury sustained by the victims or the body parts they injured. We expected that injuries associated with slips, trips, and falls, i.e., fractures, sprains, strains, and dislocations, would be more abundant in seasons when the working environment provided optimal conditions for such accidents (i.e., slippery surfaces, snow and ice formations, etc.). Slip, trip, and fall OAs can be partially prevented by using properly designed footwear [50], although this will solve only part of the problem. Another part of the solution is in improving situation awareness. This means identifying the unevenness or other faults of the level terrain (frequently hidden at a glance), following the proper procedures of machine ingress and egress [49], and using other preventive measures.

The diverse and dangerous material agents affecting forestry workers showed in the high frequency of fatal accidents. Similar results were reported by [1,51,52,53]. Limiting direct interaction between the worker and forest exterior and dangerous hand tools, such as the chain saw, proved to be a good way of decreasing the number of severe and fatal accidents in forest harvesting [53,54,55,56]. The mean share of forestry on the total number of fatalities in Slovakia was 9% (Figure 5). Considering that only about 1% of the Slovak workforce is employed in the sector, this share is high. Young workers with limited experience, who are prone to suffer fatal OAs, would likely benefit from safety seminars and courses that would help them develop suitable skills for safe logging [42]. Aside from insufficient experience in safe logging practices, Newman et al. (2018) [24] identify low situation awareness as the cause of many fatalities. In their study, they identified factors such as pressure to be highly productive at all costs, sleep deprivation, and physical fatigue, as well as the inadequate safety training, as causes of low situation awareness.

The results of the GLM analysis (Table 2) proved that the differences between the number of OAs suffered by FSR employees and contractors were statistically significant for all severity categories. The total number of reported accidents could suggest that the occupational safety of contractors was better than that of employees. However, we must keep in mind that contractors were not obliged to report minor and registered OAs. In fact, Thelin (2002) [56] states that contractors and small-scale foresters are hesitant to report safety incidents. A sign of this behavior could be found in the fact that contractors recorded fewer nonfatal accidents, but more fatal accidents, which are virtually unconcealable and must be reported to the authorities. In case of severe and fatal accidents, we attribute the significance of the difference between the number of OAs to the fact that FSR outsources the most dangerous occupations, mainly those connected to forest operations (e.g., forest harvesting, road constructions and maintenance, processing timber at conversion depots, etc.) where the likelihood of a severe OA occurring is higher than for office staff. This assumption is partially reinforced by the results of the logistic regression (Table 7), where, aside from employee age and experience, operations and occupations that occur in the field correlate significantly with the number of OAs in all severity categories. These include foresters, haulage truck drivers, and other machine operators (e.g., those used in road construction and maintenance). The data shown in Table 7 relate only to FSR employees; we were unable to conduct these analyses for contractors, as there was very little variance in the occupations (they were almost exclusively contracted to perform tasks in forest harvesting) and operations (most OAs suffered by contractors occurred during felling) they performed. On the other hand, data from other analyses (Table 1) show that contractors were indeed more likely to suffer a severe or fatal OA during their work.

This brings us to common weaknesses of studies, such the reliability of statistical data. Even though large companies like the FSR implement strong mechanisms that secure relatively reliable accident reporting, their contractors omit implementing such measures, either deliberately or because they lack the knowledge of the legislation and the skills required [39,57,58]. Furthermore, the coding of ESAW variables requires considerable skills. As Jacinto et al. (2016) [58] state there is a great interrater variability of variable coding. On the other hand, we based our analyses on a robust dataset, which contained long-term data, and coped with the problem of statistical data reliability by double-checking all OA records. The fact that the Slovak OA record contains legacy sections containing narrative descriptions of the OA helped us greatly.

## 5. Conclusions

Forestry and operations connected to forest harvesting are connected to a high risk of an occupational accident occurring. The occupational safety improved in the FSR directly over time, which can be seen in the decreased incidence rate of its employees. Outside the FSR, the number of accidents occurring also decreased; however, the rate was not as quick as for the decrease of incidence rate inside FSR. The company should, therefore, increase its efforts in enforcing the same safety measures for contractors and employees.

We found that there is a moderate to strong relationship between the volume of harvested timber and the number of severe and fatal accidents suffered by contractors in forest harvesting. Furthermore, contractors also substantially suffer more fatal accidents than employees. We also found that the incidence rate was rather uniform throughout the year. Occupational accidents at the FSR tend to occur to either young and inexperienced workers or to older workers who are all prone to decreased situation awareness. The number of occupational accidents in forestry can be reduced by further mechanizing forest harvesting, ensuring the workers (both employees and independent contractors) have sufficient knowledge of safe practices at work, and incentivizing them to work safely.

## Figures and Tables

**Table 1:** Number of occupational accidents suffered by workers according to their relationship with Forests of the Slovak Republic (FSR).

Relationship with FSR		Number of Occupational Accidents			
	Total	Registered	Minor	Severe	Fatal
E ^a^	2787	2365	265	139	18
C ^b^	153	0	0	101	52
F ^c^	23	0	0	6	17

^a^ Employees of FSR; ^b^ Contractors; ^c^ People who harvested timber allotments in FSR forests.

**Table 2:** Results of the Generalized Linear Model analysis of differences in v_2_ (number of occupational accidents) suffered by FSR employees and contractors categorized according to accident severity.

Coefficients	Estimate	Standard Error	z-value	Pr ^a^ (>|z|)
Fatal Occupational Accidents				
Intercept	0.2877	0.2700	1.065	0.287
FSR Employee	−4.7125	0.5709	−8.255	<2 × e^−16^ ***
Severe Occupational Accidents				
Intercept	−0.9163	0.2958	−3.098	0.00195 **
FSR Employee	−1.9045	0.3785	−5.032	4.86 × e^−07^ ***
Registered Occupational Accidents				
Intercept	−1.7918	0.3819	−4.692	2.71 × e^−06^ ***
FSR Employee	4.4088	0.4387	10.049	<2 × e^−16^ ***

Significance codes: 0 ‘***’ 0.001 ‘**’ 0.01; ^a^ Probability.

**Table 3:** Regression and correlation analysis of the relationship between the annual volume of harvested timber, share of salvage fellings (%), and the severity of the occupational accidents.

Factor	Severe E ^a^	Fatal E ^a^	Severe C ^b^	Fatal C ^b^
Share of incidental felling	*p* > 0.05	*p* > 0.05	*p* > 0.05	*p* < 0.05; *R* ^c^ = 0.58
Annual felling volume	*p* < 0.05; *R* = 0.55	*p* > 0.05	*p* < 0.05; *R* = 0.81	*p* < 0.05; *R* = 0.61

^a^ Employees of the forests of the Slovak Republic, GOE; ^b^ contractors; ^c^ coefficient of correlation.

**Table 4:** Type of injury sustained by the workers according to season.

Type of Injury	Winter	Spring	Summer	Fall
Strains, sprains, and dislocations	19%	24%	33%	23%
Fractures	32%	24%	25%	18%
Burns and frostbite	20%	40%	20%	20%
Multiple injuries	28%	14%	34%	24%
Concussions, internal injury	33%	0%	50%	17%
Lacerations, superficial injuries	30%	21%	28%	22%
Other specific injuries	28%	28%	20%	24%
Unknown	50%	0%	50%	0%
Head	0%	0%	33%	67%
Traumatic amputations	0%	0%	0%	100%
Mean	29%	22%	28%	21%

**Table 5:** Body parts injured of workers according to season.

Injured Body Part	Winter	Spring	Summer	Fall
Lower extremities	26%	26%	30%	17%
Upper extremities	25%	19%	29%	27%
Torso	35%	24%	15%	26%
Whole body	31%	25%	44%	0%
Head	36%	19%	19%	26%
Multiple body parts	33%	13%	40%	13%
Neck and back	27%	18%	18%	36%
Unspecified	50%	0%	50%	0%
Mean	29%	22%	28%	21%

**Table 6:** Share of occupational accidents sorted by the occupation of workers and the operations during which the accidents occurred.

Occupation	Share (%)	Operation	Share (%)
Feller	28.0	Felling	22.5
Forester	16.3	Repairs and maintenance	16.5
Mechanic	13.1	Timber haulage	12.5
HT ^a^ driver	11.9	Forest management	11.1
WPL ^b^ operator	7.1	Skidding	10.8
Forest workers	6.6	Wood processing	8.3
Other	3.5	Walking	7.8
Skidder operator	3.3	Other	3.3
WPL ^b^ supervisor	2.0	Operating other machines	1.5
Machine operator	2.0	Sideline operations ^a^	1.5
Silviculture worker	1.3	Silviculture	1.3
Animal handler	1.3	Game	1.3
Harvester operator	1.0	Animal care	0.8
Administrative	1.0	Construction and maintenance of forest roads	0.8
Helicopter pilot	0.8		
Loader operator	0.8		
Sum	100		100

^a^ Haulage truck; ^b^ wood processing line.

**Table 7:** Results of the logistic regression of the Generalized Linear Models analyzing the relationship between v_2_ (number of occupational accidents) and the v_7_ (age), v_8_ (occupation), v_9_ (experience), and v_15_ (operation) carried out by the FSR employees.

Coefficients	Estimate	Standard Error	z-Value	Pr ^a^ (>|z|)
Fatal Occupational Accidents				
(Intercept)	−10.22964	5.02516	–2.036	0.04178 *
v_9_ Experience (10–15 years category)	3.26811	1.64212	1.990	0.04657 *
v_15_ Operation (game management)	5.41262	1.92655	2.809	0.00496 **
v_15_ Operation (felling)	2.90448	1.74465	1.665	0.09595 .
Age	0.07413	0.08906	0.832	0.40523
Severe Occupational Accidents				
(Intercept)	−7.208	1.767	–4.081	4.49 × e^-5^ ***
v_9_ Experience (15–20 years category)	5.883	1.852	3.177	0.00149 **
v_9_ Experience (20–25 years category)	4.932	1.541	3.201	0.00137 **
v_7_ Age (41–50 years category)	−3.060	1.421	–2.153	0.03131 *
v_8_ Occupation (forester)	2.530	1.407	1.798	0.07219 .
v_8_ Occupation (other machine operator)	6.391	2.170	2.945	0.00323 **
v_8_ Occupation (haulage truck driver)	2.275	1.126	2.020	0.04341 *
Registered Occupational Accidents				
(Intercept)	4.2949	0.5792	7.415	1.21 × e^-13^ ***
v_7_ Age (51–60 years category)	−1.2400	0.6736	–1.841	0.0657 .
v_9_ Experience (20–25 years category)	−1.8943	0.7598	–2.493	0.0127 *
v_8_ Occupation (other machine operator)	−1.8366	1.1808	–1.555	0.1199

Significance codes: 0 ‘***’ 0.001 ‘**’ 0.01 ‘*’ 0.05 ‘.’ 0.1 ‘ ’ 1. ^a^ Probability.
